# Circadian oscillatory transcriptional programs in grapevine ripening fruits

**DOI:** 10.1186/1471-2229-14-78

**Published:** 2014-03-25

**Authors:** Pablo Carbonell-Bejerano, Virginia Rodríguez, Carolina Royo, Silvia Hernáiz, Luis Carlos Moro-González, Montserrat Torres-Viñals, José Miguel Martínez-Zapater

**Affiliations:** 1 Instituto de Ciencias de la Vid y del Vino (ICVV), Consejo Superior de Investigaciones Científicas-Universidad de La Rioja-Gobierno de La Rioja, Madre de Dios 51, 26006 Logroño, Spain; 2 Departamento de Genética Molecular de Plantas, Centro Nacional de Biotecnología (CNB), Consejo Superior de Investigaciones Científicas, Darwin 3, 28049 Madrid, Spain; 3 Bodegas Matarromera, Ctra. San Bernardo s/n, 47359 Valbuena de Duero, Valladolid, Spain; 4 Bodegas Torres S. A., Miquel Torres Carbó 6, 08720 Vilafranca del Penedès, Barcelona, Spain

**Keywords:** Circadian, Fruit ripening, Gene expression, Grapevine, Light, Microarray, Phenylpropanoid, Temperature, Terpene, *Vitis vinifera*

## Abstract

**Background:**

Temperature and solar radiation influence *Vitis vinifera* L. berry ripening. Both environmental conditions fluctuate cyclically on a daily period basis and the strength of this fluctuation affects grape ripening too. Additionally, a molecular circadian clock regulates daily cyclic expression in a large proportion of the plant transcriptome modulating multiple developmental processes in diverse plant organs and developmental phases. Circadian cycling of fruit transcriptomes has not been characterized in detail despite their putative relevance in the final composition of the fruit. Thus, in this study, gene expression throughout 24 h periods in pre-ripe berries of Tempranillo and Verdejo grapevine cultivars was followed to determine whether different ripening transcriptional programs are activated during certain times of day in different grape tissues and genotypes.

**Results:**

Microarray analyses identified oscillatory transcriptional profiles following circadian variations in the photocycle and the thermocycle. A higher number of expression oscillating transcripts were detected in samples carrying exocarp tissue including biotic stress-responsive transcripts activated around dawn. Thermotolerance-like responses and regulation of circadian clock-related genes were observed in all studied samples. Indeed, homologs of core clock genes were identified in the grapevine genome and, among them, *VvREVEILLE1* (*VvRVE1*), showed a consistent circadian expression rhythm in every grape berry tissue analysed. Light signalling components and terpenoid biosynthetic transcripts were specifically induced during the daytime in Verdejo, a cultivar bearing white-skinned and aromatic berries, whereas transcripts involved in phenylpropanoid biosynthesis were more prominently regulated in Tempranillo, a cultivar bearing black-skinned berries.

**Conclusions:**

The transcriptome of ripening fruits varies in response to daily environmental changes, which might partially be under the control of circadian clock components. Certain cultivar and berry tissue features could rely on specific circadian oscillatory expression profiles. These findings may help to a better understanding of the progress of berry ripening in short term time scales.

## Background

The grapevine fruit is a characteristic berry consisting of an external skin surrounding a fleshy pulp that encloses seeds. Grape biochemical composition is crucial for the different uses of grapes such as winemaking, production of juice and liqueur, fresh consumption or elaboration of raisins, and the final composition is mostly achieved during the ripening phase [1]. Grape ripening is triggered once the seeds have developed and radically changes fruit features from frugivore-repulsive to -attractive. This shift comprises sugar accumulation in the vacuoles of mesocarp cells accompanied by organic acid metabolisation and titratable acidity reduction [2]. Mechanisms to protect seeds from biotic and abiotic stress sources are also activated, mainly in the berry skin [3]. Phenolic compounds are accumulated, including phytoalexins, damaging light-absorbing compounds and animal-attractive anthocyanin pigments [4]. Changes in terpenoid composition result in attractive aromatic profiles, while other compounds of the same family accumulate to accomplish protective functions [5,6].

Although grapevine ripening is regulated by an intrinsic program that is partially triggered by hormonal signals [7], the process is also strongly modulated by external factors that influence the final berry composition and its commercial quality. Temperature is a major factor altering grape ripening with high temperatures hastening organic acid metabolisation and inhibiting anthocyanin accumulation [8,9]. Irradiation intensity and quality perceived in the berry skin produces changes in secondary metabolism. Light promotes flavonols and terpenoids accumulation with some effects being more specifically related with ultraviolet radiation, which is also able to enhance stilbenoids accumulation [6,10-13]. Moreover, temperature and light conditions cyclically fluctuate under field environments due to the Earth's rotation and, in fact, the strength of their daily oscillation has been shown to affect grape ripening. For instance, reduction in temperature fluctuation intensity hastens berry ripening and alters flavonoid partitioning [14] and high temperatures applied during night-times are also able to reduce anthocyanin accumulation [15]. In contrast, light pulses applied during the night-time enhance anthocyanin accumulation [16]. These effects of environmental variation on grape ripening rely, at least in part, on changes at the level of gene expression [17-21]. Thus, it can be hypothesised that daily oscillations in factors such as temperature, light or humidity could influence grape ripening progression and consequently on its final composition by means of conditioning circadian fluctuations on the grape transcriptome.

An internal molecular clock is another daily cycling element modulating plant physiology. Core clock components are transcription factors that reciprocally regulate their expression resulting in characteristic circadian expression profiles. Altogether, they determine daily rhythms of expression in a great proportion of plant transcriptomes [22-24]. In addition, the circadian clock integrates inputs from fluctuating conditions like light and temperature, which allows for plants to discriminate daily and seasonal changes [25,26]. In this way, the circadian clock together with environmental factors control several plant physiological and developmental outputs to be triggered at the proper time of day or season such as photosynthesis, starch and nitrogen metabolism, growth, cold acclimation, bud dormancy, flowering time, tuber formation or stomata and organ movements [27-29]. Since grape ripening is modulated by light and temperature inputs, it is possible that part of this modulation could be integrated by circadian clock components as shown in other plant developmental processes. However, the role of circadian clock on fruit ripening control has scarcely been considered before [30].

In this study, NimbleGen grapevine whole genome microarrays were used to follow transcriptome variations in ripening grapes throughout a 24 h daily cycle. Tempranillo and Verdejo cultivars were analysed as genotypes appreciated in Spanish viticulture for the production of red and white quality wines, respectively. Verdejo is a cultivar with different berry features than Tempranillo bearing white skin colour and a characteristic aromatic profile, giving rise to wines distinguishable from other white wines [31,32]. Given that berry flesh and skin ripening transcriptional programs significantly differ [3], and assuming that this divergence could be more pronounced in cultivars bearing black-skinned berries accordingly with the presumably higher gene expression activity related to the phenylpropanoid pathway in its berry skin, both pericarp tissues were separately analysed in Tempranillo to be more precise. This experiment was carried out using potted plants grown under controlled greenhouse conditions that allowed for identifying tissue-shared as well as tissue-specific cyclic expression profiles under monitored variation of environmental parameters. Part of this response was confirmed in a different experiment in Verdejo berries grown under field environmental conditions despite divergences in genotype, berry colour, berry total soluble solids (TSS) or sampling day photocycles and thermocycles.

## Results

### Circadian oscillatory gene expression profiles in Tempranillo berries grown under controlled conditions

**Figure 1:**
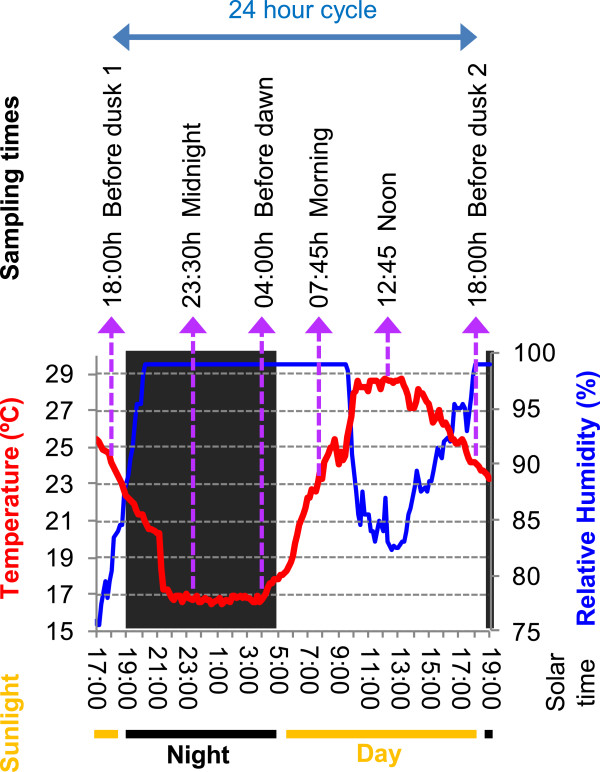
**Tempranillo berry ripening conditions and sampling set up.** Graphical representation of the evolution of temperature, ambient humidity and light conditions measured in the greenhouse throughout the time lapse of the experimental sampling (2011/08/09 and 2011/08/10 dates). Tempranillo berries were harvested at the six time points throughout a 24 hours period, as indicated. Sunlight is depicted according to sunrise and sunset times.

The question of whether sub-daily changes in environmental conditions produce specific transcriptome shifts that could influence grapevine berry ripening was addressed. Alterations putatively related with changes in metabolism taking place at the last steps of berry ripening, in the absence of earlier processes like berry softening that are more related to the veraison stage, were analysed. For this purpose, pre-ripe berries of Tempranillo (19.3 ± 0.4 °Bx) were collected at six different time points throughout a 24 h period under a controlled oscillation of light, temperature and humidity conditions. Sampling time points were selected to include times of maximum and minimum temperatures and the end of light and dark periods (12°C-difference between maximum and minimum temperature and ~10 hours of dark period during the sampling day) in order to maximize differences in the environmental conditions (Figure 1).

**Figure 2:**
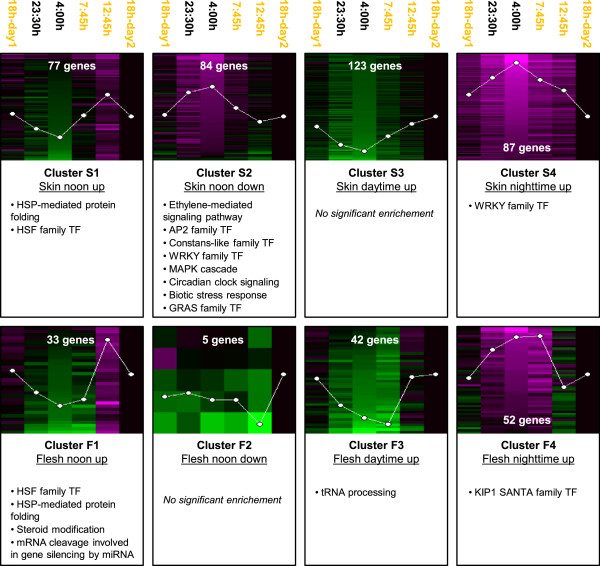
**Clustering and functional analysis of Tempranillo skin and flesh DEG throughout a 24 h daily cycle.** Significant transcripts identified for each tissue (5% FDR in Limma and ≥2-fold change) were clustered by SOMs. Four main expression profiles were identified in the skin (clusters S1 to S4) as well as in the flesh (clusters F1 to F4) of Tempranillo berries. Log_2_ expression normalized to the last time point is represented within each cluster; no difference of expression is represented in black, higher expression in magenta and lower expression in green. Number of genes within each cluster is written in white. Time points in the light period are indicated in yellow. A summary of over-represented functional categories (5% FDR) ordered by their significance level is indicated for each cluster profile.

Global transcriptome changes were first separately followed in berry skin and flesh. After applying a 5% false discovery rate (FDR) in Limma and 2-fold change cut-offs over normalized expression data on each tissue (Additional file 1), a higher number of differentially expressed genes (DEG) was identified in the berry skin (371 transcripts) than in the flesh (132 transcripts, Additional file 2). Four main expression profiles were observed in both analysed tissues, which involved oscillatory patterns following inflexion points determined by temperature or light presence variations. They included transcripts up-regulated at 12:45 pm (clusters S1 and F1 in skin and flesh, respectively), transcripts down-regulated at 12:45 pm (cluster S2 and F2), transcripts up-regulated during the daytime (clusters S3 and F3), and transcripts up-regulated during the night-time (cluster S4 and F4). Night responses were maintained until the early morning (7:45 am) in flesh clusters F3 and F4 (Figure 2). In both berry tissues, responses to heat were activated at 12:45 pm as indicated by the significant over-representation of ‘HSP-mediated protein folding’ and ‘HSF family transcription factor (TF)’ functional categories in clusters S1 and F1 (Additional file 3). Other functional categories were enriched only in specific flesh or skin clusters. Noticeably, among them, is the enrichment of ‘Ethylene-mediated signalling pathway’, ‘Circadian clock signalling pathway’, ‘Biotic stress response’, and ‘MAPK cascade’ categories as well as of AP2, Constans-like, WRKY and GRAS families of TFs in skin cluster S2. Gene silencing by miRNA was specifically enriched in cluster F1. Thus, daily light and temperature variations are related with the detection of gene expression oscillations in the skin and the flesh of pre-ripe berries, whereas genes showing oscillating expression were more frequent in the skin than in the flesh.

In order to search for those transcripts showing similar expression fluctuation in both pericarp tissues, a similar analysis (5% FDR in a 6-class Limma and 2-fold change) was run considering skin and flesh samples in the same time point as replicates. In that way, 224 DEG were identified, however, this analysis could underestimate transcripts similarly oscillating in expression in both tissues but involving differences in absolute expression levels. This result indicates that a great proportion of daily oscillating transcripts were shared between both pericarp tissues (Additional file 2). In fact, four expression profiles were identified for pericarp DEG (SF1 to SF4) that resembled those identified in individual tissues and shared with them enriched functional categories (Additional file 4). Cluster SF1 grouping transcripts up-regulated at 12:45 pm in both pericarp tissues was enriched in ‘HSP-mediated protein folding’ and ‘HSF family TF’ and moreover in ‘Vacuolar malate transport’ due to the presence of two transcripts putatively coding for tonoplast dicarboxylate transporters (*VIT_00s0187g00130* and *VIT_00s2188g00010* in Additional file 2). On the other hand, cluster SF2 grouping transcripts down-regulated at 12:45 pm in both pericarp tissues was enriched in ‘JAZ family TF’; while transcripts in cluster SF4, up-regulated in the pericarp during the night-time, included an over-representation of ‘Circadian clock signalling pathway’ as well as in KIP1 SANTA, GRAS and Constans-like families of TFs (Additional files 3 and 4). Thus, a considerable number of transcripts showed similar circadian expression oscillation in grape skin and flesh, including responses to temperature and light cycles as well as circadian clock-related genes.

### Transcripts differentially oscillating in expression between Tempranillo berry flesh and skin

**Figure 3:**
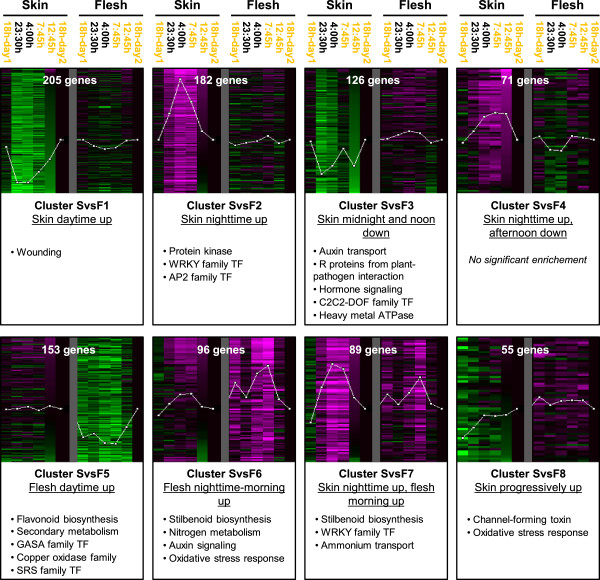
**Clustering and functional enrichment of transcripts differentially oscillating in expression between Tempranillo skin and flesh.** Transcripts differentially expressed between berry tissues (5% FDR in maSigPro ≥2-fold change) were clustered in a 4x2 SOM analysis. Log_2_ expression normalized to the last time point in the corresponding tissue is represented for each cluster. Within each tissue, no difference of expression is represented in black, higher expression in magenta and lower expression in green. Number of genes within each cluster is written in white. Time points in the light period are indicated in yellow. A summary of over-represented functional categories (5% FDR) ordered by their significance level is indicated for each cluster profile.

Once shown that berry skin and flesh shared daily expression changes in Tempranillo, tissue specific oscillations in expression in berry flesh or skin were searched by a direct comparison of both tissues. The circadian series on each Tempranillo berry tissue were compared in a two-class maSigPro time series analysis, identifying 977 DEG between tissues (5% FDR in maSigPro and 2-fold change, Additional file 5). Among them, more transcripts were specifically oscillating in expression in the skin than in the flesh (Figure 3) confirming results of analyses performed on each tissue (Figure 2). Four different skin-specific (clusters SvsF1 to 4) and two flesh-specific (clusters SvsF5 and SvsF6) oscillatory expression profiles were identified. Moreover, transcripts that were up-regulated during the night-time in both tissues but showing inductive profile maintained until the early morning only in the flesh were grouped in cluster SvsF7. Finally, transcripts showing an increased expression throughout the 24 h sampling cycle specifically in the skin were grouped in cluster SvsF8. The most abundant expression profiles were those including transcripts up-regulated specifically in the berry skin either during the daytime (cluster SvsF1) or the night-time (cluster SvsF2). Cluster SvsF1 was significantly enriched in ‘Wounding’ functional category (Additional file 6). In addition, this cluster included transcripts encoding phenylpropanoid biosynthetic enzymes like a ferulate 5-hydroxylase (*VIT_17s0000g03940*) and an anthocyanidin glycosylase (*VIT_12s0134g00590*); terpenoid biosynthetic enzymes including a vinorine synthase (*VIT_01s0010g02320*) and two β-amyrin synthase (*VIT_09s0054g01390* and *VIT_09s0054g01440*); and *MYB* TFs (*VIT_01s0026g01910*, *VIT_14s0060g00240* and *VIT_14s0006g01620*). Cluster SvsF2 was enriched in ‘Protein kinase’, ‘WRKY family TF’ and ‘AP2 family TF’ functional categories. Cluster SvsF3 included transcripts whose expression was down-regulated specifically in the skin at time points in the middle of dark and light periods. Notably this cluster is enriched for ‘R proteins from plant-pathogen interaction’ and ‘Auxin transport’ categories, the last one resulting from the presence of five transcripts encoding putative auxin transporters. Heavy metal responsive transcripts and C2C2-DOF family TFs were also enriched within this cluster.

Interestingly, the group of transcripts up-regulated during the daytime only in the flesh (cluster SvsF5) was enriched in ‘Secondary metabolism’ classified transcripts, mainly related to the presence of flavonoid biosynthetic transcripts including one phenylalanine ammonia-lyase (*VIT_13s0019g04460*), three chalcone synthase (*VIT_14s0068g00920*, *VIT_16s0022g01140* and *VIT_16s0022g01190*), 13 flavonoid hydroxylases (*VIT_06s0009g02810*, *VIT_06s0009g02830*, *VIT_06s0009g02840*, *VIT_06s0009g02860*, *VIT_06s0009g02880*, *VIT_06s0009g02920*, *VIT_06s0009g02970*, *VIT_06s0009g03010*, *VIT_06s0009g03040*, *VIT_06s0009g03050*, *VIT_06s0009g03110*, *VIT_09s0002g01090* and *VIT_16s0098g00860*) and two flavonol synthases (*VIT_02s0012g00410* and *VIT_13s0047g00210*). Other phenylpropanoid biosynthetic genes and two monoterpenoid biosynthetic transcripts coding for 10-geraniol hydroxylase (*VIT_02s0012g02820* and *VIT_03s0097g00460*) were also found in cluster SvsF5, together with *VvMybA1* (*VIT_02s0033g00380*) and *VvMybA3* (*VIT_02s0033g00450*) TFs; the former being a key regulator promoting anthocyanin biosynthesis in grapevine [33-35]. Some of these transcripts underwent a net increase in expression in the flesh during the studied 24 h period (Figure 3).

‘Stilbenoid biosynthesis’ was prominently over-represented in both clusters that involved profiles with expression peaking in the flesh at the early morning. This was due to the presence of a set of 21 stilbenoid biosynthetic transcripts among transcripts more specifically up-regulated in the flesh (cluster SvsF6) and another set of 21 transcripts within the group of transcripts that peaked earlier in the skin at the end of the night-time (cluster SvsF7). In this manner, 42 out of 45 stilbenoid biosynthetic genes annotated in the microarray were differentially involved in these two expression profiles and all of them were located in two gene clusters at chromosomes 10 and 16 (Additional file 5), which suggest a functional diversification of grapevine stilbene synthase (STS) promoters before the extensive duplication of these genes. Cluster SvsF6 was also enriched in ‘Nitrogen metabolism’ and ‘Auxin signalling’ resulting both from the presence of seven nitrilase encoding transcripts. An *IAA31-like* (*VIT_05s0020g01070*) and an auxin-responsive D22 (*VIT_05s0020g04680*) encoding transcript were also included within this profile. The enrichment of ‘Oxidative stress response’ in cluster SvsF6 was determined by the presence of six laccases, one lactoylglutathione lyase and one glutathione S-transferase. Concerning cluster SvsF7, it was enriched in ‘WRKY family TF’ and ‘Ammonium transport’ and included other phenylpropanoid biosynthetic transcripts in addition to *STSs* and the *VvMYB14* TF (*VIT_07s0005g03340*) (Additional file 5). Finally, cluster SvsF8 was enriched in transcripts belonging to the ‘Oxidative stress response’ category. Therefore, circadian gene expression patterns in the flesh and the skin of pre-ripe berries include both tissue-shared and tissue-specific oscillatory genes that in several cases might be related to specific tissue functions.

### Circadian oscillatory gene expression profiles in Verdejo berries grown under field conditions

**Figure 4:**
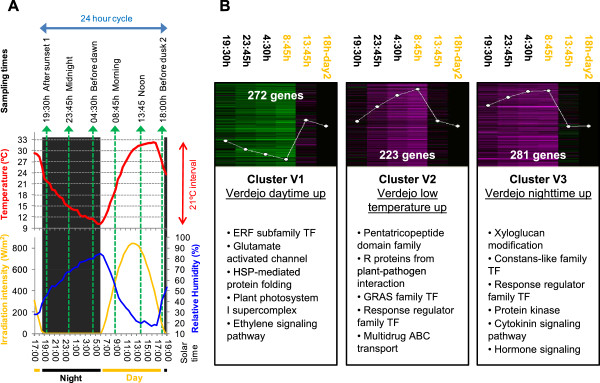
**Verdejo experimental conditions throughout the 24 h sampling cycle, clustering of Verdejo pericarp DEG and functional enrichment analysis. A**. Verdejo berry sampling set up. Graph indicating temperature, irradiation and relative humidity conditions measured in a Valbuena de Duero station near the Verdejo vineyard throughout the time lapse of the experimental sampling (2011/09/12 and 2011/09/13 dates). Verdejo berries were harvested at six time points spread throughout 24 h as specified. **B**. Clustering and functional enrichment analysis. Significant transcripts identified (5% FDR in Limma and ≥2-fold change) were clustered in a 3x1 SOM analysis. Log_2_ expression normalized to the last time point is represented within each cluster; no difference of expression is represented in black, higher expression in magenta and lower expression in green. Number of genes within each cluster appears in white. Time points in the light period are indicated in yellow. A summary of over-represented functional categories (5% FDR) ordered by their significance level is indicated for each cluster profile.

A parallel circadian experiment was set up on field grown cultivar Verdejo plants to test whether comparable daily transcriptome changes would take place in different cultivars and under vineyard conditions. Environmental conditions were slightly different during the sampling day at the Verdejo vineyard. Temperature showed a wider oscillation (21°C of interval), dark periods were slightly longer (11:30 h) and relative humidity was lower (Figure 4A). Therefore, although differential expression detected between Tempranillo and Verdejo could not be undoubtedly explained, at least those shared expression profiles could be considered as consistent cycling responses. As an important proportion of circadian regulated genes showed the same oscillatory expression profile either in Tempranillo berry skin or flesh, the analysis of Verdejo whole pericarp from pre-ripe berries (21.1 ± 0.1 °Bx) was considered suitable to be compared.

A total of 778 genes significantly oscillating in expression were identified in Verdejo berry pericarps (5% FDR in Limma and 2-fold change; Additional file 7). Almost one third of Verdejo DEG (240 transcripts) was also significant in any of the comparisons carried out in Tempranillo (Additional file 8) indicating that an important part of this response was shared between experiments regardless of genotypic, environmental and developmental differences.

When Verdejo DEG were clustered, only three main expression profile groups were detected because profiles of transcripts up-regulated around noontime could not be distinguished from those up-regulated during the daytime contrasting with the Tempranillo experiment. Such discrepancy could result from differential oscillation in environmental conditions as berries experienced the maximum temperature near noontime in the Tempranillo experiment and close to the end of the daytime in the Verdejo one (Figures 1 and 4B). Moreover, putative responses to night conditions were maintained until the early morning in Verdejo pericarp as observed for Tempranillo berry flesh (Figure 4B). The number of transcripts up-regulated during the daytime in Verdejo was almost half to that of transcripts up-regulated during the night-time. One expression profile included all daytime up-regulated transcripts (cluster V1). Similarly to Tempranillo transcripts up-regulated at 12:45 pm, cluster V1 was enriched in ‘HSP-mediated protein folding’; however it also showed enrichment in ‘ERF subfamily TF’ and ‘Plant photosystem I supercomplex’ among other categories (Additional file 9). Noticeably, cluster V1 included a *HY5*-like bZIP TF (*VIT_04s0008g05210*, Additional file 7). Concerning night up-regulated transcripts in Verdejo pericarp, the absence of light in the first time point of this experiment made it possible to discriminate between transcripts putatively up-regulated by low temperatures (cluster V2) or by darkness (cluster V3). Functional categories over-represented among transcripts up-regulated in Tempranillo berries during the night-time were also enriched in these two Verdejo clusters such as ‘GRAS family TF’ in cluster V2 and ‘Constans-like family TF’ in cluster V3. Additionally, cluster V2 was enriched in ‘Biotic stress response’ and ‘ABC multidrug transport’. Two putative 1-deoxy-D-xylulose 5-phosphate synthase (DXS) encoding transcripts were present within cluster V2 (*VIT_11s0052g01730* and *VIT_11s0052g01780*), while the first one was also induced during the night-time in Tempranillo berry skin (cluster SvsF2) together with another putative *DXS* (*VIT_09s0002g02050*). Verdejo cluster V3 was enriched in ‘Xyloglucan modification’ because it contained eight xyloglucan endotransglucosylase (XET) encoding transcripts (Additional file 7). ‘Cytokinin-mediated signalling pathway’ was also over-represented in cluster V3, which included three transcripts coding for authentic-response regulator (ARR) type-A (*VIT_08s0007g05390*, *VIT_13s0067g03430* and *VIT_13s0067g03480*) and one for histidine kinase *AHK2*-like cytokinin receptor (VIT_12s0057g00690), while ‘Response regulator family TF’ was enriched in both V2 and V3 clusters of night-time up-regulated transcripts also due to the presence of several *ARR* genes. Noticeably, *VvGAI1* (*VIT_01s0011g05260*), encoding a repressor of gibberellin responses [36], was also grouped within the V3 cluster.

Although differential expression profiles between Tempranillo and Verdejo could not be clearly attributable to differences in genotype or growth conditions, time series in both cultivars were explicitly compared. Considering that Tempranillo berry skin and flesh shared circadian oscillatory gene expression (Additional file 2) and that a considerable portion of the ripening transcriptional program is common to both pericarp tissues [3], Tempranillo skin and flesh samples on each time point were considered as replicates to be compared to Verdejo pericarp samples. In this manner, 286 DEG were identified (5% FDR in a maSigPro and 2-fold change, Additional file 10). More transcripts specifically oscillating in expression were identified in Verdejo than in Tempranillo. However, they were enriched in similar functional categories as those regulated in Tempranillo. They involved ‘HSP-mediated protein folding’ and ‘Response regulator family TF’ among transcripts up-regulated only in Verdejo during the daytime or during the night-time, respectively (clusters TvsV3 and 4 in Additional files 11 and 12). Apart from these similarities, differences in secondary metabolism were observed. Three caffeoyl-CoA O-methyltransferase (*VIT_01s0010g03470*, *VIT_01s0010g03490* and *VIT_01s0010g03510*) and one secoisolariciresinol dehydrogenase (*VIT_06s0004g07540*) phenylpropanoid biosynthetic transcripts were up-regulated during the daytime only in Tempranillo (cluster TvsV1). Another secoisolariciresinol dehydrogenase (*VIT_08s0058g00790*) and a flavonoid hydroxylase (*VIT_18s0001g11520*) were induced during the night-time specifically in Tempranillo (cluster TvsV2). In contrast, terpenoid biosynthetic transcripts specifically oscillated in expression in Verdejo including a linalool synthase (*VIT_00s0271g00060*), a myrcene synthase (*VIT_00s0271g00030*), two β-amyrin synthase (*VIT_10s0003g03520* and *VIT_10s0003g03650*) and three lupeol synthase (*VIT_10s0003g03600*, *VIT_10s0003g03610* and *VIT_10s0003g03620*) that were up-regulated during the daytime; while an isoprene synthase (*VIT_12s0134g00020*), a β-ocimene synthase (*VIT_12s0134g00030*) and a germacrene synthase (*VIT_18s0001g04560*) were up-regulated during the night-time. Interestingly, *VvMYBF1* (=*VvMYB12*, *VIT_07s0005g01210*), a light inducible TF [19,37], was up-regulated during the daytime only in the Verdejo experiment (Additional file 10). These results indicate that circadian expression changes in grapes are mostly consistent among different genotypes. Divergences putatively related with genotypic or environmental differences were also found.

### Circadian clock homologous genes oscillating in expression in grapevine berries

Given that expression profiles enriched in circadian clock signalling-related functional categories were identified in all analysed datasets, homologs to core clock components within the grapevine 12× V1 genome gene predictions were searched for, in order to analyse their expression profiles throughout the studied 24 h cycles (Table 1). *VvLHY* (*VIT_15s0048g02410*) was the only identified grapevine ortholog to *Arabidopsis thaliana CCA1* and *LHY* partially redundant morning core clock MYB genes. This indicates that the *LHY* duplication observed in other Angiosperms took place after the separation of clades giving rise to the Vitaceae as has previously been reported [38]. *VvLHY* did not show any significant oscillation in expression in the experiments contrasting to the results obtained for another homolog to morning MYB TFs, *VvRVE1* (*VIT_04s0079g00410*). This grapevine gene was previously annotated as CIR1/RVE2 (Additional file 2), despite it is the closest homolog to Arabidopsis *REVEILLE1* (*RVE1*) circadian clock gene [39]. Transcription of *VvRVE1* was up-regulated during the night-time and peaked in the morning in Tempranillo and Verdejo pericarps (Additional files 3 and 9). In addition, *VvPPR7_1* (*VIT_06s0004g03650*) was up-regulated during the daytime in Tempranillo pericarp, while *VvELF3_2* (*VIT_09s0002g02680*) expression peaked in the morning in Verdejo berries. Several grapevine homologs to night core clock genes could be identified in the grapevine genome including *VvTOC1* (*VIT_17s0000g06570*), although they did not significantly change in expression. Therefore, core circadian clock gene homologs are present in the grapevine genome and only some of them seem to oscillate in expression in berry tissues.

**Table 1:** Grapevine closest homologues to Arabidopsis core clock proteins

**Protein**	**ID**	**Length (aa)**	**Query cover (%)**	**Identity**^**a **^**(%)**	**Significant in cluster**^**b**^
CCA1 (AT2G46830, 608 aa)					
VvLHY	VIT_15s0048g02410	771	100	40	No significant
VvRVE1	VIT_04s0079g00410	479	39	71	S4, SF4, V2
LHY (AT1G01060, 645 aa)					
VvLHY	VIT_15s0048g02410	771	100	44	No significant
VvRVE1	VIT_04s0079g00410	479	23	79	S4, SF4, V2
RVE1 (AT5G17300, 387 aa)					
VvLHY	VIT_15s0048g02410	771	39	54	No significant
VvRVE1	VIT_04s0079g00410	479	92	45	S4, SF4, V2
PRR7 (AT5G02810, 727 aa)					
VvPPR7_1	VIT_06s0004g03650	482	70	52	SF3
VvPRR7_2	VIT_13s0067g03390	769	95	44	No significant
VvPPR9	VIT_15s0048g02540	641	27	55	No significant
PRR9 (AT2G46790, 468 aa)					
VvPPR9	VIT_15s0048g02540	482	93	58	No significant
VvPRR7_2	VIT_13s0067g03390	769	60	48	No significant
PRR5 ( AT5G24470, 667 aa)					
VvPRR5	VIT_16s0098g00900	688	82	41	No significant
VvPPR9	VIT_15s0048g02540	482	76	59	No significant
TOC1 ( AT5G61380, 618 aa)					
VvTOC1	VIT_17s0000g06570	551	93	51	No significant
VvTOC1-like	VIT_17s0000g06520	129	24	55	No significant
LUX (AT3G46640, 323 aa)					
VvLUX	VIT_06s0004g05120	311	97	53	No significant
GI (AT1G22770, 1173 aa)					
VvGI	VIT_18s0157g00020	1170	99	77	No significant
ELF3 (AT2G25930, 695 aa)					
VvELF3_1	VIT_04s0008g00660	725	99	41	No significant
VvELF3_2	VIT_09s0002g02680	740	99	32	V2
ELF4 (AT2G40080, 111 aa)					
VvELF4	VIT_13s0067g00860	145	82	69	No significant

^a^Coverage of the Arabidopsis protein by the grapevine predicted protein sequence.

^b^Clusters of significant transcripts in Figures 2, 3 and 4 and Additional files 5 and 10.

## Discussion

### Berry transcriptome plasticity in the adaptation to daily environmental changes

Plants as sessile organisms have evolved physiological and developmental adaptations to changes in the surrounding environment. Grape transcriptome changes promoted by the soil composition and weather conditions of different years and locations have been described as examples of plasticity displayed by the berry ripening transcriptome [40]. Herein changes are shown in the berry ripening transcriptome taking place within the same plants as a response to sub-daily fluctuations in the surrounding environment. After applying consistent statistical analyses and cut-offs, a total of 1433 and 868 transcripts showing circadian oscillation in expression were respectively identified in Tempranillo and Verdejo experiments (Additional files 2, 5, 7 and 10). Although these daily oscillations could involve a smaller proportion of circadian cycling genes than in other species or tissues [23,41], they are larger than those transcriptome changes observed between consecutive berry ripening stages near maturity, consistently described to be low under diverse experimental conditions and genotypes [3,42,43]. Thus, sub-daily variation of gene expression in the berry could be more decisive for the final berry composition than other developmental cues at late ripening stages.

Most detected cycling profiles were apparently directed to adapt berry cells to changing environmental conditions such as temperature and light. They included the over-expression of *HSPs*, *ERF* TFs and *BAG5*-like Bcl-2 associated athanogene (*VIT_01s0146g00150*) positively correlating with high temperatures as well as the down-regulation of ABC transporters expression negatively correlating with them as described for thermotolerance responses in Muscat Hamburg berries [17]. The identification of a higher number of *HSPs* specifically induced during the daytime in Verdejo berries, which experienced more extreme temperatures than the Tempranillo ones, also suggests the existence of plasticity in berry thermotolerance responses. Nonetheless, some of these as well as other circadian transcriptional responses observed herein may not result in physiological responses as they might not always involve changes at the protein level [44].

On the other hand, transcriptional changes related to biotic stress responses were activated during the night-time and more greatly in samples including berry skin. Induction of pathogen *R*-gene-mediated resistance before dawn, the time when the pathogen normally disperses the spores, has been described as a mechanism controlled by core clock genes in Arabidopsis to anticipate infection [45,46]. An analogous mechanism in the grape berry, an organ particularly susceptible to pathogen attacks when approaching ripeness, could have been evolutionarily selected to ensure seeds dispersal and species survival. TFs of the WRKY family were also induced around dawn in all analysed datasets suggesting that they could participate in the regulation of this defence response [47,48]. *STSs* were simultaneously induced only in Tempranillo skin and flesh. Higher expression of *STS* transcripts has also been correlated with low temperatures in Corvina berries grown under different environmental conditions [40]. Circadian regulation of *STS* genes could be partially controlled by *VvMYB14,* which was also induced at dawn only in Tempranillo berry skin, since this TF is able to activate *STS* promoters in grapevine [49]. In contrast, activation of defence pathways against virus at warmest times of day could be characteristic of grape berries in view that RNA silencing *ARGONAUTE2* homologues (*VIT_10s0042g01150* and *VIT_10s0042g01180*) were induced around noontime in all experiments. In fact, plant RNA silencing-mediated defence pathways are generally less active under low temperatures and more active at high temperatures concomitantly with plant virus activity [50,51].

*CONSTANS*-like genes participate in the integration of light and circadian clock signalling [25,52], whereas transcripts within this family consistently peaked around dawn both in Tempranillo and Verdejo berries. Relative to circadian clock signalling, *CCA1* and *LHY* are considered the core morning loop genes in the Arabidopsis molecular clock [53-55]. *VvLHY* was the only ortholog to these genes found in the grapevine reference genome and did not oscillated in expression in our experiments (Table 1). In contrast, in all analysed grape samples, *VvRVE1* showed a cyclic expression profile paralleling these of *LHY*/*CCA1* genes in Arabidopsis leaves. This gene is the ortholog of *RVE1*, another MYB CCA1 subfamily circadian clock-related gene that, like LHY and CCA1, is able to bind to the ‘evening element’ in the promoter of evening-phased circadian rhythmic genes [56,57]. Taking into account these coincidences, it might be interesting to test whether *VvRVE1* could act as a core clock morning gene in grapevine pre-ripe fruits. Absence of significant regulation in other central clock homologs in the grape analyses may suggest that a simplified version of the clock would operate in grapevine late ripening fruits as shown in other non-leaf Arabidopsis organs like roots [58]. The biology of grapevine fruits is extremely different to that of plant tissues and species where the circadian clock signalling has been explained. Therefore, further research is required to confirm how distinct can this pathway be in particular grapevine tissues. On the other hand, hormone signalling was enriched within several expression profiles peaking in the morning in Tempranillo and Verdejo berries. Hormonal responses coordinated by light- and circadian clock-mediated signalling were shown important for daily promotion of growth in Arabidopsis [59]. Thus, it might be worth testing whether similar mechanisms could modulate grape growth and ripening processes that are also under the control of phytohormones [7,60]. For instance, relative to gibberellins signalling, rhythmic expression of *DELLA* genes peaking around dawn under circadian clock control has been described in Arabidopsis seedlings [61] and similar expression profile of *VvGAI1* was observed in Verdejo pericarp.

### Berry composition features affected by daily changing conditions

Final composition of mature grape berries is highly related with activity of secondary metabolism. The differential daily cycling responses between berry tissues and cultivars included greater variations in the expression of phenylpropanoid biosynthetic transcripts in the Tempranillo experiment despite these results could be underestimated in our approach. *VvMybA1* and *VvMybA3* TFs were co-induced during the daytime only in Tempranillo berry flesh together with a large group of flavonoid biosynthetic transcripts. The hybridization signal for *VvMybA1* and *VvMybA3* microarray probe sets was constitutively higher in Tempranillo berry skin and in Verdejo pericarp when compared to Tempranillo flesh samples (Additional file 1). This could indicate that cross hybridization between both TF probe sets and transcripts takes place in the microarray because, as known, *VvMybA3* should be expressed in Verdejo berries but not *VvMybA1,* whose expression is not expected in white cultivars [34,62-65]. Indeed, *VvGT1* (*VIT_16s0039g02230*), a direct target of VvMybA1 TF and considered to encode the anthocyanidin-glycosyltransferase catalysing the limiting step for anthocyanin accumulation in berry skin [33,35,66], was constitutively more highly expressed in Tempranillo berry skin than in flesh and very much less in Verdejo pericarp (Additional file 1). Further research would be required to show whether VvMybA1 or VvMybA3 positively regulate flavonoid biosynthesis in grape mesocarp cells in a circadian cycle-dependent manner.

The activation of terpenoid biosynthetic genes during the daytime, including monoterpenoid synthases, concurrently to that of light signalling components took place only in Verdejo white and aromatic berries. These light signalling genes included a homolog of *HY5*, a key gene involved in light perception transduction in Arabidopsis [67,68], and *VvMYBF1*, which is induced by sunlight and promotes flavonol biosynthesis in grapes [19,20,37]. Absolute expression of *VvMYBF1* and this *HY5* homolog (*VIT_04s0008g05210*) during the daytime was higher in Verdejo than in Tempranillo samples (Additional file 1). Photosynthetic genes activated during the daytime only in Verdejo could also be indicative of greater light responses in these berries as some of them are induced by *HY5* and by the circadian clock during the daytime in Arabidopsis [69]. Considering these results together with the fact that scent terpenoids accumulation in grapes is enhanced by light [6,12], it would be interesting to test whether HY5 and VvMYBF1 TFs regulate light induced terpenoid biosynthesis in grapes. Additionally, the extension of circadian gene expression analysis to other black and white cultivars under different cycles of controlled temperature and irradiation conditions would help to explain if some of these specific responses are related to the experimental conditions, the berry skin colour or to other cultivar-specific features.

Concerning primary metabolism-related ripening processes, breakdown of malic acid in grapes is hastened by high temperatures [8,17,70]. Only a few genes related to this process cycled in expression despite great temperature oscillations took place during the sampling time courses. Interestingly, two dicarboxylate vacuolar transporters, with a likely function in organic acids import into the vacuole [71], were induced at 12:45 pm in Tempranillo berries opposing high temperature effects on malic acid metabolisation. In general, results suggest that circadian changes of expression in grapes at late ripening stages affect more deeply secondary than primary metabolism pathways. Part of these expression changes may have important consequences on the final berry composition including berry features depending either on the plant genotype or on the circadian fluctuations in the surrounding environment.

## Conclusions

Our study demonstrates the existence of daily oscillatory changes in the grapevine berry transcriptome at late ripening stages. These expression profiles involved responses to temperature and light fluctuation as well as oscillation of circadian clock components. Identified expression profiles suggest that regulatory genes such as a *VvRVE1* circadian clock homologous gene as well as Constans-like, WRKY and GRAS family TFs might promote night-activated processes in all analysed experiments. Evidence of circadian variations in the expression of these elements in pre-ripe grapes was shown for the first time in this study. Certain tissue-specific oscillatory expression profiles were related to berry skin and flesh features. Circadian expression profiles in secondary metabolism-related transcripts may impact on the progression of ripening and the final composition of berries, which could result in part from the cyclic expression of chief regulatory genes like *VvMybA1*, *VvMybA3*, *VvMYBF1, VvMYB14* or a *HY5* homolog. In summary, our results could contribute to understanding the progress of berry ripening over sub-daily time scales in response to circadian environmental changes. These findings could lead to new considerations in models directed to predict the effect of ripening season environmental conditions on the final berry composition.

## Methods

### Plant material

Experiment using *V. vinifera* cv. Tempranillo was conducted on three years old potted plants, grafted on rootstock 110 Richter. Soil inside pots (50 · 45 · 40 cm) was composed by a peat:sand:vegetal sand mix (63:25:12) over a 5 cm stones layer at the bottom. Tempranillo plants were housed in an experimental greenhouse from Bodegas Miguel Torres S.A. located in Pacs del Penedès (Catalonia, Northeast Spain). Greenhouse was roof-covered with glass allowing for sunlight transmission and no other light was supplied. Total irradiation across the glass was reduced in 44 ± 6% (mean ± SD; Additional file 13). Pots were covered with white plastic films and dropper irrigated according to plant requirements that were calculated as: week water supply = WU * PET * watering coefficient (WU, water use by plants in the previous week; PET, potential evapotranspiration; watering coefficient, 90%). WU was calculated by using weighing lysimeters. Temperature inside the chamber was regulated by Exafan coolers (Grup Sabater, Argentona, Spain) to be the same as external ambient temperature. Temperature and humidity inside the chamber were registered every 10 min by MCU Clima sensors (Grup Sabater). Vines were pruned during the vegetative phase and shoot apexes were trimmed throughout the ripening season. Three separated blocks of ten plants inside the greenhouse were used as biological replicates for the Tempranillo experiment.

Experiment using *V. vinifera* cv. Verdejo was conducted with five years old vines from a grapevine cultivars garden collection belonging to Bodegas Matarromera S.L. located in Valbuena de Duero (Valladolid, Castilla y León, central plateau Spain). Verdejo vines grafted on 110 Richter rootstocks were conducted in a driving trellis system, in a row orientated in E-W direction. The Verdejo vineyard was dropper irrigated twice throughout the ripening season (2 L · h^−1^) and defoliation practises were not carried out. Three blocks of ten plants in the same row separated by other ten plants in between were delimited as biological replicates for the Verdejo experiment. For this experiment, meteorological data from Valbuena de Duero station obtained from SiAR website (http://eportal.magrama.gob.es/websiar/ were considered.

### Experimental design and berry sampling

To identify berry gene expression changes taking place during a daily cycle, grapes were harvested at six time points throughout a 24 hour period. Time point selection was aimed at maximizing differential environmental conditions: 1, dusk; 2, midnight; 3, before dawn; 4, at the early morning; 5, at the time of maximum temperature (near noontime) and 6, before dusk of the second day. A pre-maturity stage was selected for the sampling day to analyse expression changes at the late ripening phase. Berries from three different bunches were harvested for each sample. On each cultivar, berries of the same density were selected to homogenize the ripening state among all sampled time points and blocks. To do this, every berry was separated from its cluster by cutting the pedicel and its density was determined by floatability in a NaCl solution series as a non-invasive indication of the internal sugar concentration [72-74]. The density interval showing higher berry abundance just before the 24-hours sampling cycle onset was selected for every sample of the same cultivar. TSS in berries from the selected density interval was measured by a digital refractometer WM-7 (ATAGO, Tokyo, Japan). For the Tempranillo experiment, berries between 110–130 g NaCl · l^−1^ (corresponding to a mean ± SD TSS between replicates of 19.3 ± 0.4 °Bx) were collected at 2011/08/09, 18:00 and 23:30 as well as at 2011/08/10 4:00, 7:45, 12:45 and 18:00 time points (Figure 1). For the Verdejo experiment berries between 130–150 g NaCl · l^−1^ (corresponding to a mean ± SD TSS of 21.1 ± 0.1 °Bx) were collected at 2011/09/12, 19:30 and 23:45 as well as at 2011/09/12 4:30, 8:45, 13:45 and 18:00 time points (Figure 4). Verdejo berries were always obtained from clusters collected from the north side of the vines. The first time point differed between experiments since it was just before dusk for Tempranillo and after the beginning of the dark period for Verdejo. In all cases solar time is indicated, which corresponds to two hours less than local official time. Sampling took place during completely sunny days in the Verdejo experiment and with only some high clouds in the second afternoon of the Tempranillo experiment. Density selected berries were immediately rinsed in distilled H_2_O, frozen in liquid nitrogen and kept at -80°C until further analyses. Time lapses from cluster collection to berry freezing were between 10 to 20 min.

### Gene expression analyses

#### RNA isolation

Tempranillo and Verdejo frozen berries were peeled and de-seeded, respectively. About 10–15 berries were used for RNA extraction on each sample. Total RNA was extracted from frozen Tempranillo berry skin and flesh and Verdejo pericarp as described by Reid et al. [75]. DNase digestion of contaminating DNA in the RNA samples was carried out with the RNase-Free DNase Set (QIAGEN). Final RNA purification was carried out using the Spectrum™ Plant Total RNA kit (Sigma-Aldrich) according to standard protocols.

#### Microarray hybridization and data processing

RNA integrity of each RNA preparation was tested using an Agilent 2100 Bioanalyzer (Agilent technologies). cDNA was synthesized from 10 μg of total RNA using the cDNA Synthesis System Kit (NimbleGen-Roche). The cDNA preparation (1 μg) was amplified and labelled with Cy3-random nonamers using the One-Color Labeling Kit (NimbleGen-Roche). If the quality control was correct, then 4 μg of labelled cDNA were hybridized on a NimbleGen microarray 090818 Vitis exp HX12 (NimbleGen-Roche), representing 29,549 predicted genes on the basis of the grapevine 12× V1 gene prediction version (platform GPL17894 in Gene Expression Omnibus [GEO] database). Hybridization solution (NimbleGen Hybridization kit) was added to each labelled cDNA and hybridization was performed for 16 h at 42°C in a HS 4 Hybridization station (NimbleGen-Roche). Hybridized microarrays were washed with Wash buffer kit (NimbleGen-Roche) and scanned at 532 nm and 2 μm resolution in a DNA Microarray Scanner with Surescan High-Resolution Technology (Agilent technologies).

After evaluation of hybridization quality by experimental metrics report implemented in the NimbleScan software version 2.6 (NimbleGen-Roche), probe set signal values from all the microarray hybridizations were background corrected and normalized together using the robust microarray average (RMA) [76] in the NimbleScan software, which produces calls file for each sample with normalized expression data for each gene. The full microarray expression data are available on GEO under the accession number GSE52167. A dataset was generated from normalized data including the expression of the 29,549 annotated genes in the 54 analysed samples (Additional file 1).

#### Identification of differentially expressed transcripts and functional analysis

Expression changes during a daily period were searched for each analysed series (Tempranillo berry skin, Tempranillo berry flesh and Verdejo pericarp). On each case, in order to identify transcripts showing changes of expression in any of six analysed time points, a six-class Limma comparison was carried out in Babelomics suite [77,78]. A *P* value of 0.05 after the Benjamini-Hochberg adjustment for multiple testing and a ≥ 2-fold changes between any two stages were applied as significance cut-offs on each time series. The same cut-offs were applied in Limma after considering Tempranillo skin and flesh samples for the same time point as replicates to identify transcripts with consistent oscillation in expression along the pericarp. To directly contrast gene expression oscillations in a daily period between Tempranillo berry tissues, skin and flesh series were compared using a maSigPro two-class time series comparison [79], which was also conducted in Babelomics suite. Polynomial degree 3 was selected to be able to identify transcripts cyclically oscillating in expression. *P* values of 0.05 were chosen as significance levels for gene selection after Benjamini-Hochberg correction and for model variable in maSigPro. In addition, a ≥ 2-fold difference between tissues in at least one time point after expression data normalization to the corresponding 18:00 h time point of the second day on each tissue and besides, a ≥ 2-fold change between any two stages in at least one tissue were applied. These fold change cut-offs were aimed at identifying transcripts oscillating in expression in at least one tissue that showed considerable differences in their profiles independently of differences in absolute expression between tissues. The same cut-offs were used in maSigPro to identify transcripts with differential oscillation in expression between the 24 h periods analysed in Tempranillo and Verdejo experiments. In this case, Tempranillo skin and flesh samples for the same time point were considered replicates to pick out transcripts with consistent expression along Tempranillo pericarp that was the tissue analysed in Verdejo.

DEG identified in all three Limma analysis and both maSigPro comparisons were clustered by shared expression profiles after Log_2_ expression data normalization to the expression in 18:00 h day-two time point of the corresponding tissue and cultivar using self-organizing maps (SOM) analysis [80]. Euclidean squared metrics and scaled rows were selected for the SOM that were run in Acuity 4.0 (Axon Molecular Devices, http://www.moleculardevices.com). For each SOM analysis, the most informative number of clusters was assessed by gap statistical analyses [81], which were also carried out in Acuity 4.0. Each identified cluster was analysed on Babelomics suite to search for significant functional enrichment following a grapevine specific functional classification of 12× V1 predicted transcripts [82]. Fisher’s exact test was carried out in a FatiGO analysis [83] to compare each study list to the list of total transcripts housed in the grapevine 12× V1 gene predictions [84]. Significant enrichment was considered in case of *P* value ≤0.05 after Benjamini and Hochberg correction for multiple testing. Finally lists of significant transcripts identified from different analysis were compared by Venn diagrams performed in Venny (http://bioinfogp.cnb.csic.es/tools/venny/index.html) [85].

### Search of circadian clock gene homologues

The grapevine genomic sequence was searched for loci encoding homologous proteins to Arabidopsis CCA1, LHY, PRR9, PRR7, PRR5, GI, TOC1, LUX, ELF3 and ELF3, PRR5 core clock proteins [89]. A BLAT alignment from each Arabidopsis protein sequence to the PN40024 12X genomic sequence was carried out in the Genoscope website (http://www.genoscope.cns.fr/blat-server/cgi-bin/vitis/webBlat). For each locus, the corresponding protein in the 12× V1 gene annotations version was identified from Grimplet et al. (2012). Grapevine 12× V1 protein sequences were obtained from the Uniprot website (http://www.uniprot.org/) and were aligned to Arabidopsis protein sequences by blastp (http://blast.ncbi.nlm.nih.gov/).

## Competing interests

Authors declare no competing interests.

## Authors’ contributions

PCB participated in the conception of the study, design of experiments, berry sampling and classification, analysed data and drafted the manuscript. VR obtained RNAs from frozen berries. CR and SH participated in berry sampling and classification. MTV and LCMG participated in the design of the study and maintenance of plants. JMMZ participated in the conception of the study, design of experiments and manuscript drafting. All authors critically revised the manuscript. All authors read and approved the final manuscript.

## Supplementary Material

Additional file 1 RMA normalized expression data table. Click here for file

Additional file 2 DEG throughout a 24 h cycle in Tempranillo berry skin, flesh and pericarp table. Click here for file

Additional file 3 Functional analysis in clusters of differentially expressed transcripts identified in Tempranillo skin, flesh and pericarp table. Click here for file

Additional file 4 **Clustering and functional analysis of Tempranillo pericarp DEG throughout a 24 h daily cycle figure.** Skin and flesh samples of the same time point were considered replicates to identify transcripts similarly oscillating in expression in both berry tissues (5% FDR in Limma and ≥2-fold change). Four major expression profiles were identified by SOMs (clusters SF1 to SF4). Expression normalized to 18 h day 2 is shown for each cluster; no difference of expression is represented in black, higher expression in magenta and lower expression in green. Number of genes within each cluster is written in white. Time points in the light period are indicated in yellow. A summary of over-represented functional categories (5% FDR) ordered by their significance level is indicated for each cluster profile. Click here for file

Additional file 5 Transcripts differentially oscillating in expression between Tempranillo berry skin and flesh table. Click here for file

Additional file 6 Functional enrichment analysis in clusters of transcripts differentially oscillating in expression between Tempranillo skin and flesh table. Click here for file

Additional file 7 DEG throughout a 24 h cycle in Verdejo pericarp table. Click here for file

Additional file 8 **Venn diagrams comparing Tempranillo and Verdejo DEG Figure. A**. Venn diagram comparing lists of significant transcripts (5% FDR and 2-fold change) identified in Tempranillo skin (T skin), Tempranillo flesh (T flesh) and Verdejo by Limma as well as these identified between Tempranillo skin and flesh (T SvsF) by maSigPro analysis. A total of 211 transcripts were significant in Verdejo and in any of these Tempranillo comparisons. **B**. Venn diagram comparing the list of significant transcripts identified in Verdejo pericarp to the list of significant transcripts identified in Tempranillo (5% FDR and 2-fold change) only when skin and flesh samples were considered replicates (SF specific). Additional 29 transcripts oscillating in expression in Verdejo were identified by this analysis in Tempranillo. Click here for file

Additional file 9 Functional enrichment analysis in clusters of differentially expressed transcripts identified in Verdejo pericarp table. Click here for file

Additional file 10 Transcripts differentially oscillating in expression between Tempranillo and Verdejo experiments table. Click here for file

Additional file 11 **Clustering and functional enrichment of transcripts differentially oscillating in expression between Tempranillo and Verdejo experiments figure.** Transcripts differentially expressed between 24 h cycles analysed for each cultivar (5% FDR in maSigPro ≥2-fold change) were clustered in a 4x1 SOM analysis. Tempranillo skin and flesh samples for the same time point were considered replicates. Log_2_ expression normalized to the last time point in the corresponding cultivar is represented for each cluster. Within each cultivar, no difference of expression is represented in black, higher expression in magenta and lower expression in green. Number of genes within each cluster is written in white. Time points in the light period are indicated in yellow. A summary of over-represented functional categories (5% FDR) ordered by their significance level is indicated for each cluster profile. Click here for file

Additional file 12 Functional enrichment analysis in clusters of transcripts differentially oscillating in expression between Tempranillo and Verdejo experiments table. Click here for file

Additional file 13 Irradiation intensity inside the Tempranillo greenhouse. Click here for file
