# Supplementary figures and images for: Circadian oscillatory transcriptional programs in grapevine ripening fruits
Source: BMC Plant Biol. 2014 Mar 25;14:78. doi: 10.1186/1471-2229-14-78 (PMC3986946; doi:10.1186/1471-2229-14-78)

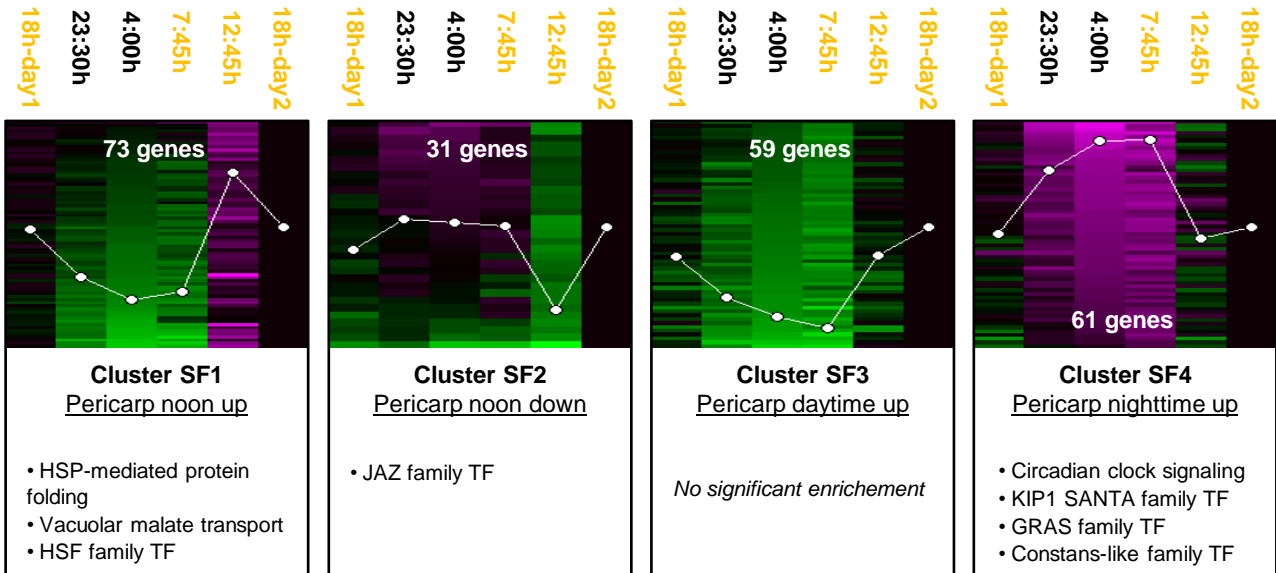

Supplement: Additional file 4 — Clustering and functional analysis of Tempranillo pericarp DEG throughout a 24 h daily cycle figure. Skin and flesh samples of the same time point were considered replicates to identify transcripts similarly oscillating in expression in both berry tissues (5% FDR in Limma and ≥2-fold change). Four major expression profiles were identified by SOMs (clusters SF1 to SF4). Expression normalized to 18 h day 2 is shown for each cluster; no difference of expression is represented in black, higher expression in magenta and lower expression in green. Number of genes within each cluster is written in white. Time points in the light period are indicated in yellow. A summary of over-represented functional categories (5% FDR) ordered by their significance level is indicated for each cluster profile. [file 1471-2229-14-78-S4.pdf]

**A**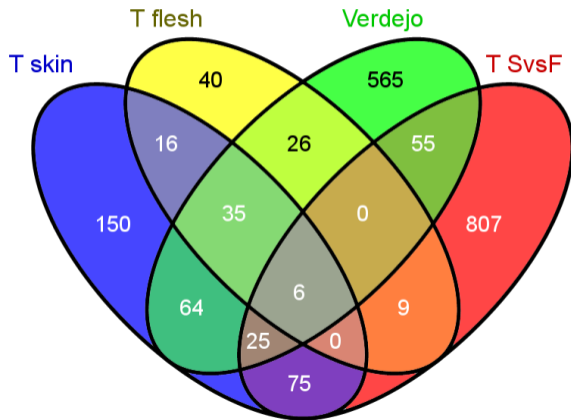**B**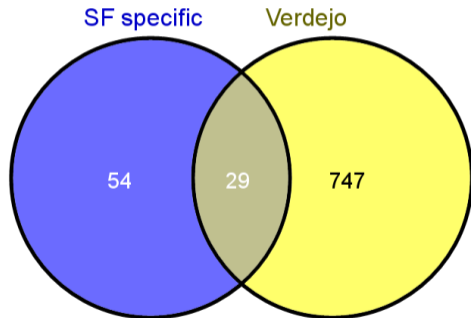

Supplement: Additional file 8 — Venn diagrams comparing Tempranillo and Verdejo DEG Figure. A. Venn diagram comparing lists of significant transcripts (5% FDR and 2-fold change) identified in Tempranillo skin (T skin), Tempranillo flesh (T flesh) and Verdejo by Limma as well as these identified between Tempranillo skin and flesh (T SvsF) by maSigPro analysis. A total of 211 transcripts were significant in Verdejo and in any of these Tempranillo comparisons. B. Venn diagram comparing the list of significant transcripts identified in Verdejo pericarp to the list of significant transcripts identified in Tempranillo (5% FDR and 2-fold change) only when skin and flesh samples were considered replicates (SF specific). Additional 29 transcripts oscillating in expression in Verdejo were identified by this analysis in Tempranillo. [file 1471-2229-14-78-S8.pdf]
