# Supplementary material for: Circadian oscillatory transcriptional programs in grapevine ripening fruits
Source: BMC Plant Biol. 2014 Mar 25;14:78. doi: 10.1186/1471-2229-14-78 (PMC3986946; doi:10.1186/1471-2229-14-78)
Supplement: Additional file 11 — Clustering and functional enrichment of transcripts differentially oscillating in expression between Tempranillo and Verdejo experiments figure. Transcripts differentially expressed between 24 h cycles analysed for each cultivar (5% FDR in maSigPro ≥2-fold change) were clustered in a 4x1 SOM analysis. Tempranillo skin and flesh samples for the same time point were considered replicates. Log2 expression normalized to the last time point in the corresponding cultivar is represented for each cluster. Within each cultivar, no difference of expression is represented in black, higher expression in magenta and lower expression in green. Number of genes within each cluster is written in white. Time points in the light period are indicated in yellow. A summary of over-represented functional categories (5% FDR) ordered by their significance level is indicated for each cluster profile. [file 1471-2229-14-78-S11.pdf]

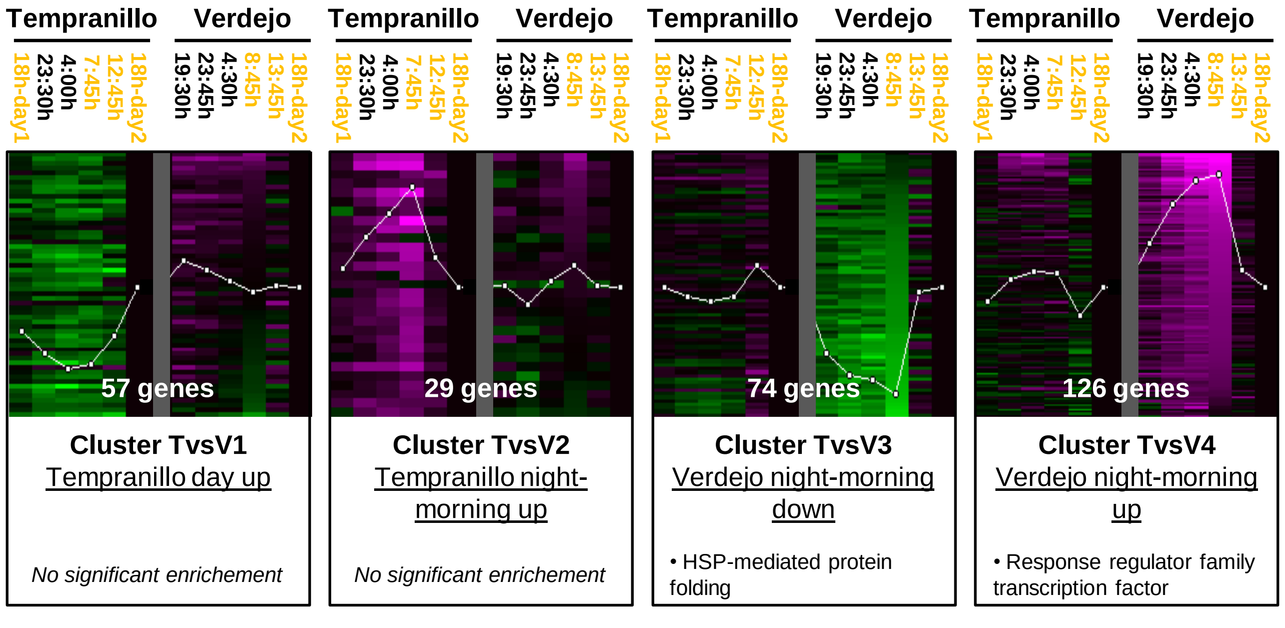

Tempranillo
Verdejo
18h-day1
23:30h
4:00h
7:45h
12:45h
18h-day2
19:30h
23:45h
4:30h
8:45h
13:45h
18h-day2
57 genes
Cluster TvsV1
Tempranillo day up
No significant enrichement
29 genes
Cluster TvsV2
Tempranillo night-morning up
No significant enrichement
74 genes
Cluster TvsV3
Verdejo night-morning down
• HSP-mediated protein folding
126 genes
Cluster TvsV4
Verdejo night-morning up
• Response regulator family transcription factor
